# SEM analysis of the tunable honeycomb structure of irradiated poly(vinyl chloride) films doped with polyphosphate

**DOI:** 10.1016/j.heliyon.2018.e01013

**Published:** 2018-12-08

**Authors:** Mohammad Hayal Alotaibi, Gamal A. El-Hiti, Hassan Hashim, Ayad S. Hameed, Dina S. Ahmed, Emad Yousif

**Affiliations:** a National Center for Petrochemicals Technology, King Abdulaziz City for Science and Technology, P.O. Box 6086, Riyadh, 11442, Saudi Arabia; b Department of Optometry, College of Applied Medical Sciences, King Saud University, P.O. Box 10219, Riyadh, 11433, Saudi Arabia; c Department of Physics, College of Science, Al-Nahrain University, Baghdad, Iraq; d Department of Chemistry, College of Science, Tikrit University, Tikrit, 34001, Iraq; e Department of Chemistry, College of Science, Al-Nahrain University, Baghdad, 64021, Iraq

**Keywords:** Materials science, Materials chemistry, Physical chemistry

## Abstract

The fabrication of tunable poly(vinyl chloride) porous films containing polyphosphate as an additive was successful. Irradiation of poly(vinyl chloride) films containing polyphosphate at a low concentration (0.5% by weight) with an ultraviolet light (λ_max_ = 313 nm) for 300 h leads to the formation of a honeycomb like structure. The scanning electron microscopy images, at different magnification power, confirmed the production of the PVC honeycomb-like structure. The morphological images of the polymeric film showed a rough surface and a large number of regularly distributed hexagonal pores. The number of pores increased upon irradiation time and it was maximum after 300 h. The honeycomb structure formation could be due to the regular aggregation of polyphosphate among the polymeric chains, the increase in solution intrinsic viscosity and evaluation of hydrogen chloride gas through dehydrochlorination process.

## 1 Introduction

Honeycomb-like materials have light weight, strength, and tailorable mechanical performances [1, 2]. They can be used as core materials in various applications, ranging from low-cost doors to advanced aerospace structures, as sandwich panels. In addition, flexible honeycomb structures have been suggested as an alternative for morphing skin [3, 4] and have potential applications in separation membranes [5], microarrays [6], anti-reflective coatings [7], transparent super-hydrophobic surfaces [8] and biosensors [9]. Honeycomb porous films can be synthesized using various techniques such the solvent casting, airflow, dip coating, spreading, spin-coating and on-water surface [10]. Several approaches have been used to modify the pores shape, such as the use of pores templates, shrinking and stretching techniques [10]. Significant progress has been made to produce tunable honeycomb structure of polymeric films [11, 12]. Nanomaterials and nanocomposites have unique physical and chemical properties such as high porosity and surface area and can be used in the selective separation and storage of gases such as carbon dioxide, methane and nitrogen, for example [13, 14, 15]. Therefore, the design and synthesis of such materials are great interest.

Poly(vinyl chloride) (PVC) is one of the world's largest production of universal plastics [16, 17]. PVC can be used in automobiles, office equipment, furniture, sidings, windows, packaging, pipes and electronic appliances [18, 19]. For PVC to be used in sustainable building construction, it should be recyclable, durable and produce low CO_2_ emission during the manufacturing process. PVC can be used in exterior sidings in new family houses. In the USA alone, in 2010, it accounted for *ca*. 36% of the total PVC use [20], mainly because of the low production cost. However, PVC suffers from photodegradation due to natural weathering factors such as light, heat and moisture. PVC dehydrochlorination commonly takes place as a result of structural defects (*e.g*. allylic chlorine, tertiary chlorine) within the polymeric chains [21, 22, 23]. Such processes cause the PVC to blacken at high temperature (100–200 °C) due to the physical and chemical changes within the polymeric materials [24]. Therefore, various additives have been used to reduce the photodegradation of PVC [25, 26, 27, 28, 29, 30, 31].

As part of our continuing research in the area of polymeric materials [32, 33, 34, 35], we became interested in the synthesis of highly ordered PVC honeycomb porous films, due to their various applications, using a simple and efficient technique. Recently, we reported a simple process for the fabrication of highly ordered PVC honeycomb films containing a low concentration of a nickel(II) Schiff base complex [36]. We now report the successful production of a well-ordered porous PVC honeycomb-like structure, using tetrahydrofuran (THF) as the solvent, while employing the casting method upon irradiation with ultraviolet light (UV) for a long period. The casting method is a simple and good technique to produce homogeneous films with a high surface area.

## 2 Experimental

**Fig. 1:**
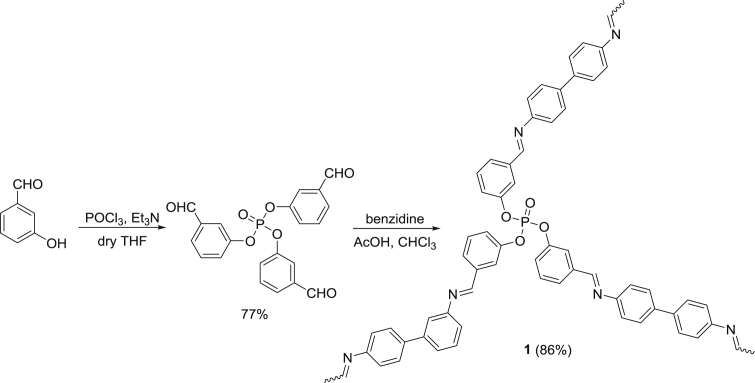
Synthesis of polyphosphate **1**.

Polyphosphate **1** was synthesized as previously reported [32]. Treatment of 3-hydroxybenzaldehyde with phosphoryl chloride in the presence of triethylamine (Et_3_N) in boiling THF for 5 h gave the corresponding *tris*(3-formylphenyl)phosphate in 77% yield which on reaction with excess benzidine (3 mole equivalents) in the presence of acetic acid (AcOH) in boiling chloroform (CHCl_3_) for 6 h gave the corresponding polyphosphate **1** in 86% yield (Fig. 1). A mixture of PVC (1 g) and polyphosphate **1** (5 mg) in THF (10 mL) was stirred for 30 min at 25 °C [37, 38, 39, 40]. The mixture was casted onto a clean glass plate (15 holes; 4 × 4 cm^2^) and dried at 25 °C for 24 h. Any residual solvent left was removed by drying the samples at 25 °C for 3 h under vacuum. The films were removed and their thickness (*ca*. 40 μm) was measured using a Digital Caliper DIN 862 micrometer (Vogel GmbH, Kevelaer, Germany). The PVC films were fixed on aluminum plates (Q-panel company, Homestead, FL, USA). The process was carried out for three times to test the consistency. The humidity was controlled during the PVC preparation.

The PVC films were irradiated with a continuous exposure to a UV light (λ_max_ = 313 nm and light intensity = 6.43 × 10^−9^ ein.dm^−3^.s^−1^) for 300 h at 25 °C using QUV accelerated weathering tester (Philips, Saarbrücken, Germany). Such technique reproduces the damage that could be caused by the direct exposure to sunlight. The morphology of the prepared PVC porous films was examined with a scanning electron microscopy (SEM) using Inspect S50 microscope (FEI Company, Czech Republic) at 15 Kv as an accelerating voltage.

## 3 Results and discussion

**Fig. 2:**
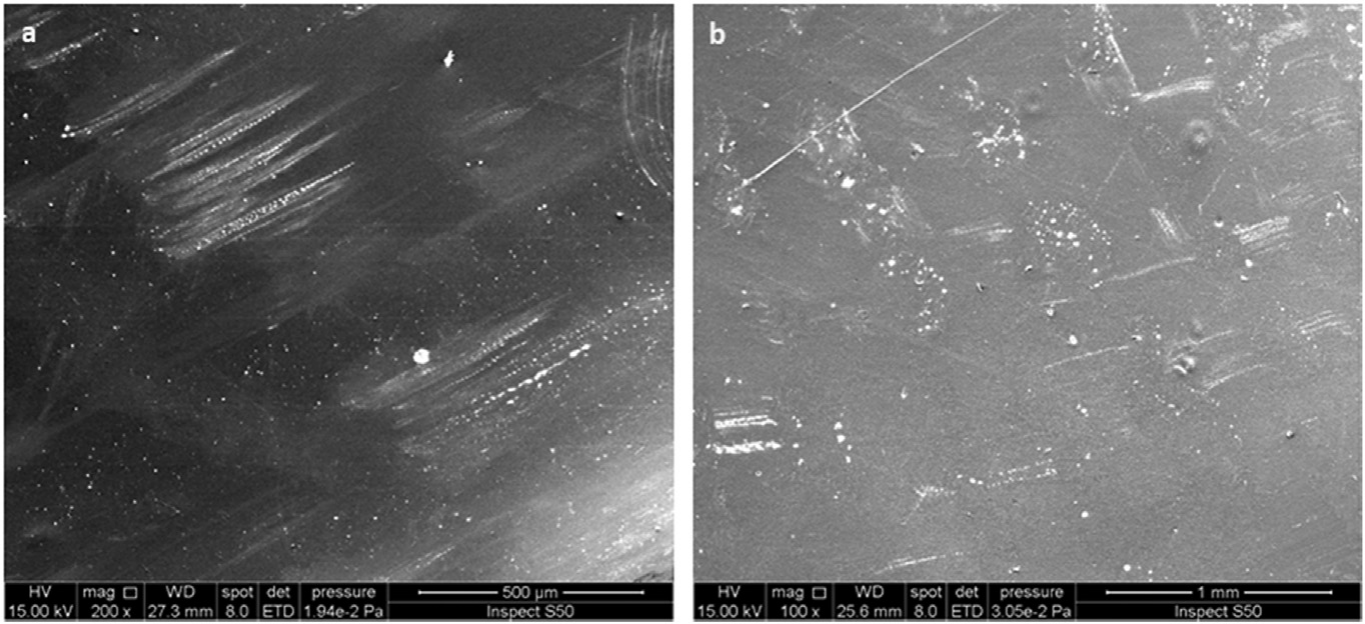
SEM images of the PVC film (blank) before irradiation: (a) (500 μm); (b) (1 μm).

**Fig. 3:**
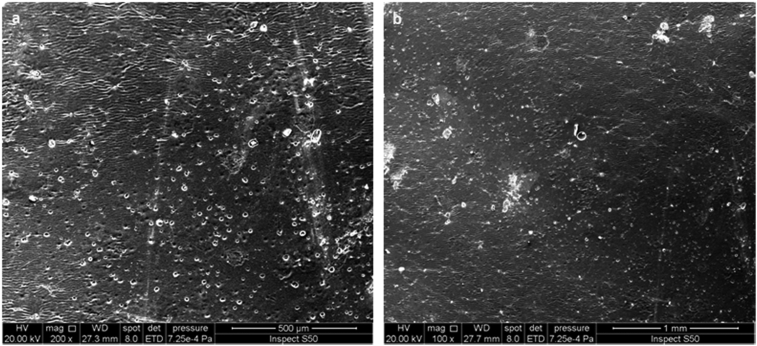
SEM images of the PVC film (blank) after irradiation (300 h): (a) (500 μm); (b) (1 μm).

The morphology of the surface of the PVC film (blank) before and after irradiation was examined by the SEM, at different magnification powers, at room temperature. The SEM images indicated that the surface of the PVC film (blank) before irradiation was generally smooth (Fig. 2). However, the SEM images recorded after irradiation (300 h) showed a damaged within the PVC surface and formation of many cracks (Fig. 3) as a result of photodegradation of polymeric chain. It was clear that no PVC porous structure was produced either before or after irradiation for the PVC film.

**Fig. 4:**
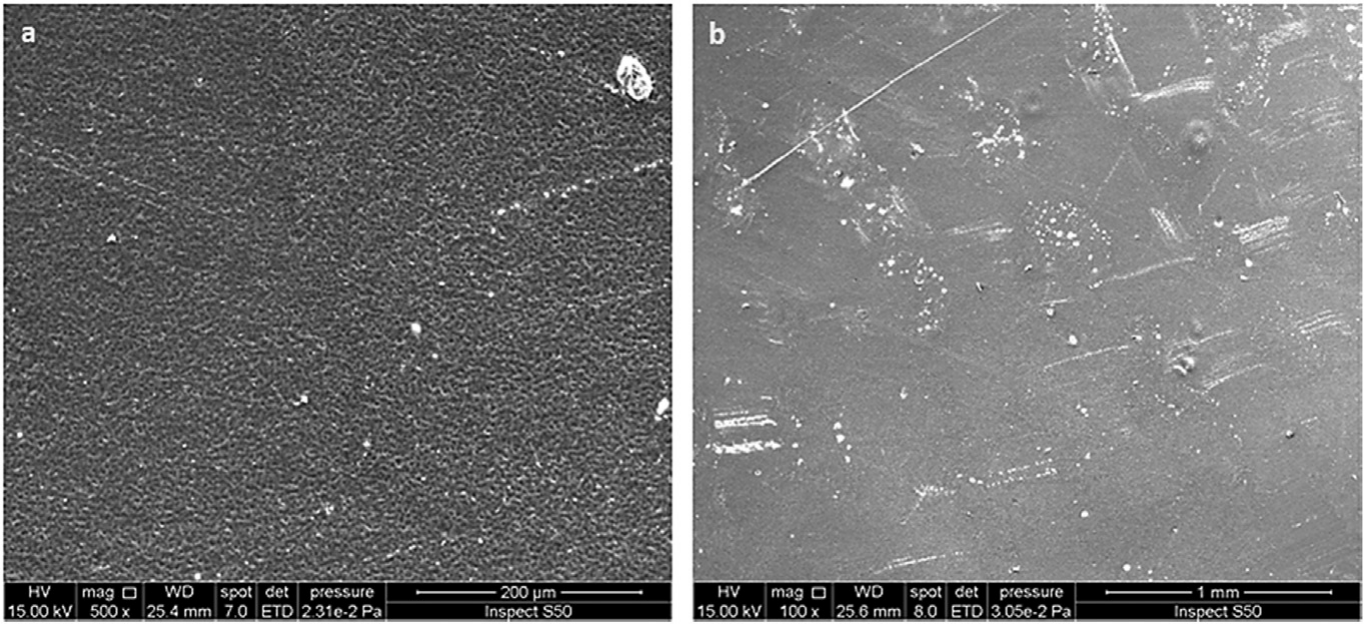
SEM images of the PVC film containing **1** before irradiation: (a) (200 μm); (b) (1 μm).

The SEM images of the surface of the PVC film containing **1** (0.5 wt%) were recorded at room temperature before and after irradiation with a UV light. The morphological features of the PVC film containing **1** before irradiation, at different magnification power (200 and 1 μm), showed that surface was smooth, neat and has no cracks with little flaws (Fig. 4). Clearly, no PVC porous structure was obtained before the irradiation process.

**Fig. 5:**
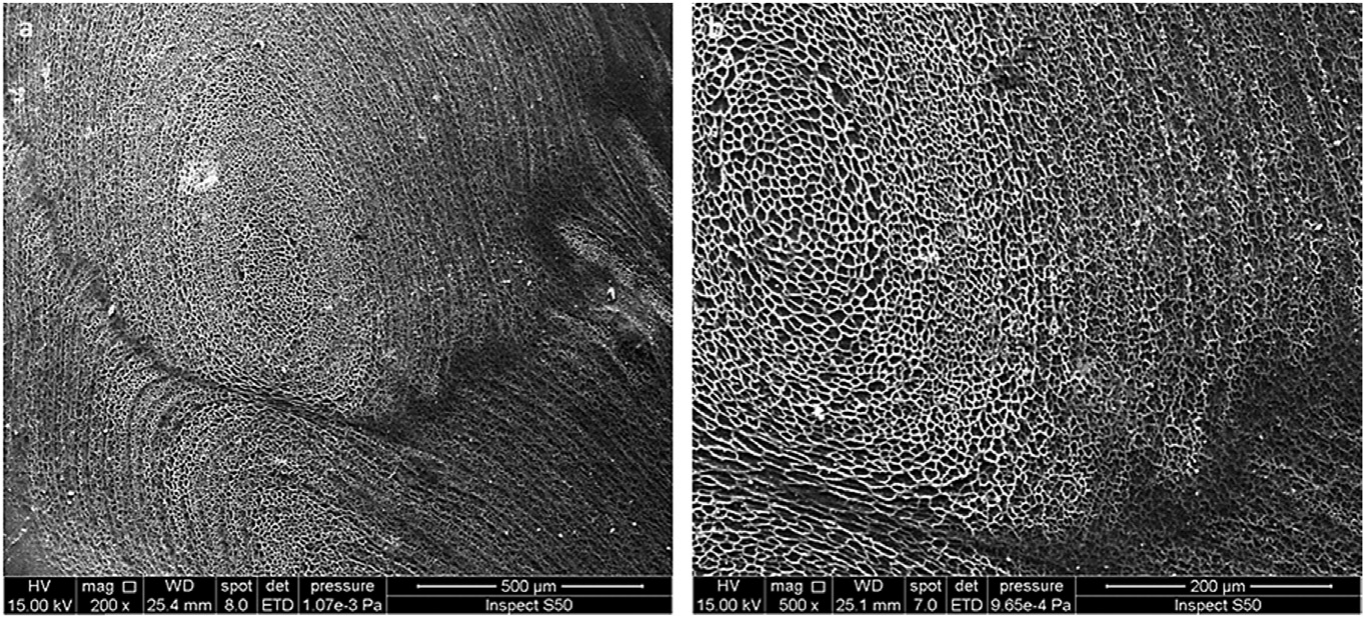
SEM images of the PVC film containing **1** after irradiation (300 h): (a) 500 μm; (b) 200 μm.

**Fig. 6:**
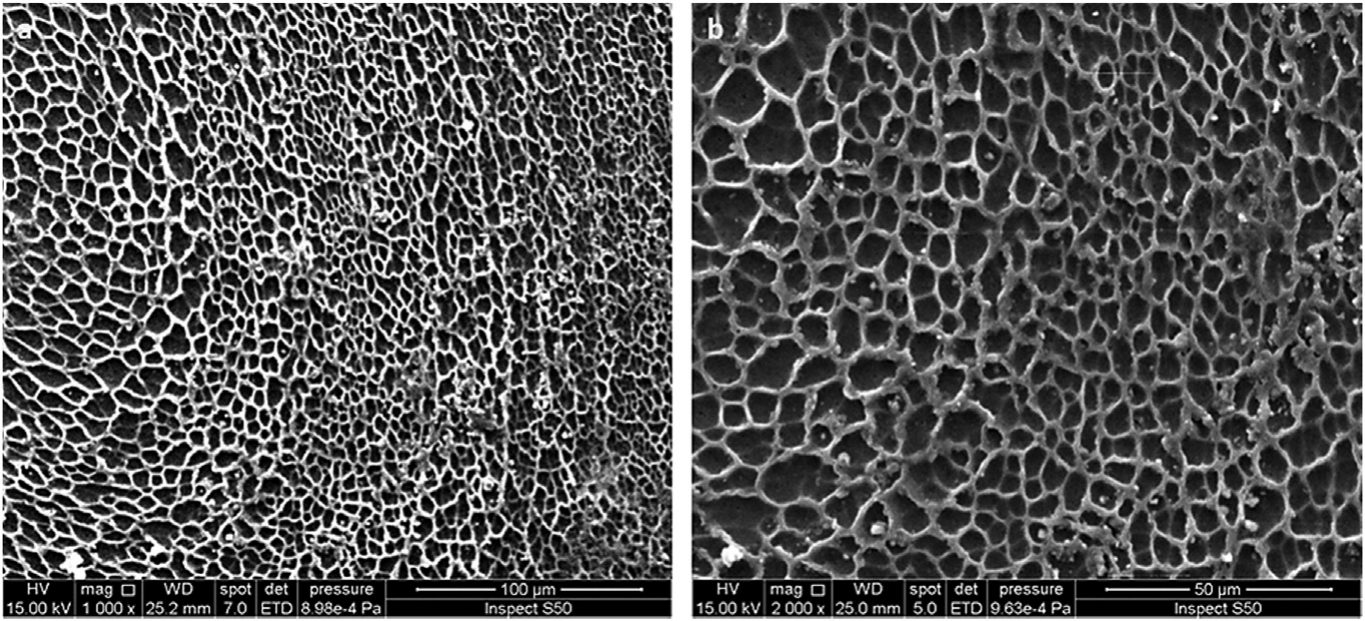
SEM images of the PVC film containing **1** after irradiation (300 h): (a) 100 μm; (b) 50 μm.

**Fig. 7:**
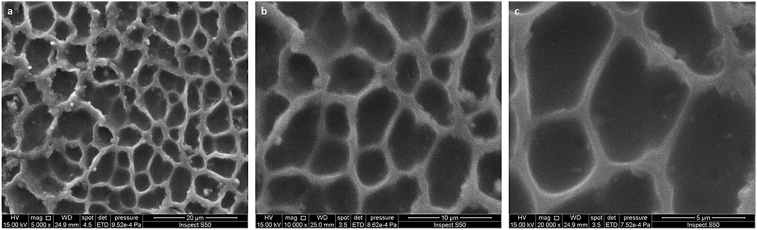
SEM images of the PVC film containing **1** after irradiation (300 h): (a) 20 μm; (b) 10 μm; (c) 1 μm.

The PVC film containing **1** was irradiated with a UV light (λ_max_ = 313 nm) for up to 300 h and the surface morphology was inspected by the SEM at different magnification power (Figs. 5, 6, and 7). Fig. 5 (500 and 200 μm width) showed that the PVC surface was rough and has a regular porous structure. A large number of pores appeared that are regularly distributed on the PVC surface. Irradiation of the PVC film for less 300 h leads to fewer numbers of holes and smaller pores size in comparison to the ones obtained when the irradiation time was 300 h. Fig. 6 (100 and 50 μm width) showed clearly a highly-ordered PVC honeycomb structure after irradiation (300 h).

Fig. 7 (20–1 μm width) showed that the pores were hexagonal in shape and each one was surrounded by six hexagonal pores which is similar to a honeycomb. The SEM micrographs showed that the PVC surface smoothness gradually decreases as irradiation time increases from 0 to 300 h, but the number of holes significantly increased.

The regular aggregation of polyphosphate **1** among the PVC polymeric chains helps porous structure formation upon irradiation. The phenomena could be due to the increase in solution intrinsic viscosity [41]. Long irradiation time could lead to apparent holes within the PVC surface. Also, the dehydrochlorination process in which hydrogen chloride gas was evolved leads to a PVC weight loss and high functional group indices due to production of small fragments that contain various functional groups [25, 26, 27, 28, 29]. In addition, photodegradation process of PVC leads to the formation of cross-linked chains which could be the reason for the honeycomb porous structure formation. Previous reports indicated that cross-linked materials are ideal for the production of honeycomb-like structures in which condensed water was stabilized [42, 43, 44, 45, 46, 47]. For example, deep irradiation (6 h at 25 °C) of crossed linked polystyrene thin film leads to the formation of a honeycomb-like structure [43]. The honeycomb film was fabricated using the phase separation method which involves the use of chloroform and methanol mixture (9:1 by volume) [43]. Also, a honeycomb film of poly(acrylic glycidyl ether) was produced upon irradiation of the polymeric material, with a UV light for a short time, in dichloromethane or chloroform as a solvent, using the breath figure technique [44].

Honeycomb porous PVC films were previously synthesized with THF as a hydrophilic solvent by the breath figures method [41]. The honeycomb structure was found to be dependent on various factors such as concentration, humidity, solvent, polymer architectures and the method adopted [42]. Moreover, the solution concentration and relative humidity play a significant role in the formation of regular honeycomb film of other polymeric materials [48, 49].

## 4 Conclusion

A well-ordered porous poly(vinyl chloride) film, containing a low concentration of polyphosphate, was fabricated using the casting method in which tetrahydrofuran was used as a solvent. The scanning electron microscopy images of the PVC film indicated the presence of a large number of hexagonal pores. It has been demonstrated that increasing irradiation time can lead to an increase in the number of pores within the PVC surface. The process is simple and efficient and could be used for the large scale production of honeycomb like structure of polymeric films. No porous PVC honeycomb structure was obtained before irradiation.

## Declarations

### Author contribution statement

Mohammad H. Alotaibi: Contributed reagents, materials, analysis tools or data.

Gamal A. El-Hiti, Hassan Hashim, Emad Yousif: Conceived and designed the experiments; Wrote the paper.

Dina S. Ahmed: Performed the experiments; Analyzed and interpreted the data.

### Funding statement

This work was supported by King Abdulaziz City for Science and Technology (KACST), Saudi Arabia (grant No. 020-0180), Tikrit and Al-Nahrain Universities.

### Competing interest statement

The authors declare no conflict of interest.

### Additional information

No additional information is available for this paper.
